# Temporal Characterization of Prion Shedding in Secreta of White-Tailed Deer in Longitudinal Study of Chronic Wasting Disease, United States

**DOI:** 10.3201/eid3010.240159

**Published:** 2024-10

**Authors:** Nathaniel D. Denkers, Erin E. McNulty, Caitlyn N. Kraft, Amy V. Nalls, Joseph A. Westrich, Edward A. Hoover, Candace K. Mathiason

**Affiliations:** Colorado State University, Fort Collins, Colorado, USA

**Keywords:** chronic wasting disease, CWD, prions, urine, saliva, feces, secreta, excreta, white-tailed deer, real-time quaking induced conversion, RT-QuIC, IPQ, prions and related diseases, United States

## Abstract

Chronic wasting disease (CWD) affects cervids in North America, Asia, and Scandinavia. CWD is unique in its efficient spread, partially because of contact with infectious prions shed in secreta. To assess temporal profiles of CWD prion shedding, we collected saliva, urine, and feces from white-tailed deer for 66 months after exposure to low oral doses of CWD-positive brain tissue or saliva. We analyzed prion seeding activity by using modified amyloid amplification assays incorporating iron oxide bead extraction, which improved CWD detection and reduced false positives. CWD prions were detected in feces, urine, and saliva as early as 6 months postinfection. More frequent and consistent shedding was observed in deer homozygous for glycine at prion protein gene codon 96 than in deer expressing alternate genotypes. Our findings demonstrate that improved amplification methods can be used to identify early antemortem CWD prion shedding, which might aid in disease surveillance of cervids.

Chronic wasting disease (CWD) has been identified in North America, Asia, and Scandinavia (*1*–*4*) and is the only known prion disease in wildlife. CWD is often considered the most infectious of all prion diseases, but how CWD spreads so efficiently remains unclear. It is generally accepted that facile CWD transmission results from robust prion replication in target tissues, leading to shedding of infectious prions into saliva, urine, and feces (i.e., secreta). Cervids are likely exposed to infectious prions via direct animal-to-animal contact or indirect contact with the agent shed into the environment.

Landscapes previously housing CWD-infected cervids are contaminated with sufficient infectious prions to initiate subsequent infections (*5*,*6*). Fomite-only exposure of uninfected deer to buckets and bedding from suites housing CWD-positive animals resulted in CWD infections in disease-naive deer within 19 months after exposure (*7*). Studies in white-tailed deer (WTD; *Odocoileus virginianus*) also revealed that large oral doses of saliva, urine, or feces contained adequate concentrations of the CWD agent to initiate infections (*8*,*9*). Additional studies in WTD established a minimum oral CWD infectious dose equivalent to 100–300 ng CWD-positive brain tissue (*10*). Yet, equivalent saliva doses (16.5–30 mL) seem large compared with a dose expected in nature. Nevertheless, taken together, those studies indicate doses of CWD prions in saliva and exposure to shed prions in the environment are sufficient for CWD transmission.

The temporal shedding profile of CWD prions in secreta remains poorly understood. Little is known about the onset and duration of the complete prion shedding profile during the asymptomatic phase of CWD infection. It is suspected that prion levels in shed secreta are low, and inhibitors or nonspecific substrate activators in secreta can constrain the use of sensitive in vitro amplification assays, such as serial protein misfolding amplification (sPMCA) and real-time quaking-induced conversion (RT-QuIC) (*11*–*14*). To enhance detection capabilities of those assays, sodium phosphotungstic acid or iron oxide bead capture techniques have been used to concentrate prions and reduce or bypass interfering factors (*15*–*26*). Thus, continued in vitro assay optimization has improved CWD antemortem detection.

In addition to the route and dose by which CWD prions are transmitted, the sequence of the prion protein gene *PRNP* is another factor affecting disease pathogenesis. The prion protein is highly conserved between cervids (*27*), although single-nucleotide polymorphisms within the gene sequence can lead to reduced susceptibility (*28*), slower disease progression (*29*,*30*), and prolonged survival (*31*,*32*), all of which might affect prion shedding (*18*,*33*). Specific codon polymorphisms known to influence cervid CWD susceptibility are codons 132 in elk (M→L) (*28*), 225 in mule deer (S→F) (*31*), and 96 in WTD (G→S) (*34*). As additional polymorphisms are identified (*35*), the role they play in CWD susceptibility, pathogenesis, and shedding will need to be explored.

We used iron oxide bead (IOB) capture in combination with sPMCA and RT-QuIC to profile longitudinal (66 months) prion shedding in urine, saliva, and feces collected from WTD exposed to low oral doses of CWD prions. Because dose and *PRNP* genotype affect initial detection of infection and disease onset, we correlated those factors with prion shedding consistency and duration. Our goal was to provide a more complete understanding of prion shedding onset and duration that contributes to CWD pathogenesis and transmission and aid in early antemortem detection by using minimally invasive methods.

## Methods

### Animals

We obtained CWD-free WTD fawns from the University of Georgia Warnell School of Forestry and Natural Resources (Athens, GA, USA). At 4 months of age, the fawns were transported to the indoor CWD research facility at Colorado State University (Fort Collins, CO, USA), where we conducted studies under strict guidelines according to protocols approved by the Institutional Animal Care and Use Committee (protocol nos. 18-8396A and 1242). We housed cohorts of deer in separate suites. We housed mock-infected control deer in different suites within the same facility; they served as sentinels to ensure that no unexpected pathogen exposures occurred during the long course of CWD prion infections.

### Inoculations

We exposed 12 WTD to low oral doses of CWD-positive brain tissue (n = 8) or saliva (n = 4); seeding activities were equivalent to 1 mg (n = 4) or 300 ng (n = 4) CWD-positive brain tissue or 300 ng (n = 4) CWD-positive saliva. Deer number, inoculum source, dose, regimen, and genotypes were described previously (*10*,*36*). Because access to WTD is limited and to conserve animals, 2 sham-inoculated deer served as negative controls. Each sham-inoculated deer received a total of 300 ng CWD-negative brain material and 300 ng CWD-negative saliva that had been preincubated with 600 mg of montmorillonite clay. We administered the sham inocula as 3 doses of 200 ng CWD-negative material (100 ng brain + 100 ng saliva + 200 mg montmorillonite clay) 1 time/week for 3 consecutive weeks.

### *PRNP* Genotype

We determined the *PRNP* genotype of the deer at codon 96 before beginning the study; 7 deer expressed 96GG and 5 expressed 96GS polymorphisms. The negative control deer were both 96GG. One 96GG deer also had a rare polymorphism at codon 103 (103NT). In an unrelated study (N.D. Denkers, unpub. data), we identified another deer expressing codon 103NT; both deer had disease courses similar to deer expressing the 96GS polymorphism. Because a previous low-dose study demonstrated no observable clinical differences (*10*), we compared animals with wild-type codon 96GG genotypes (cohort 1; n = 6) with deer expressing *PRNP* gene polymorphisms 96GS or 103NT that delayed CWD infection (cohort 2; n = 6). Cohort 1 consisted of 5 male and 1 female deer; cohort 2 had 3 male and 3 female deer.

### Sample Collections

We performed tonsil and recto-anal mucosa-associated lymphoid tissue biopsies at 3 months postinoculation (mpi) and analyzed the tissue for amyloid seeding activity by using RT-QuIC and for CWD prion protein (PrP^cwd^) deposition by using immunohistochemistry. We considered deer to be infected with CWD prions when 2 consecutive tonsil biopsies were RT-QuIC positive (*36*).

We collected saliva, urine, and feces in conjunction with lymphoid biopsy tissue (Table 1). We collected urine from male deer for 21 months by free catch, then manually by bladder expression. We catheterized female deer and obtained urine via syringe aspiration. We collected saliva by syringe aspiration of the buccal pouch. We collected feces directly from the rectum and placed it into specimen cups. We collected all samples by using clean single-use syringes or containers, which we then aliquoted and stored at –80° C until analysis.

**Table 1:** Summary of collected tissues and secreta in longitudinal study of temporal characterization of prion shedding in white-tailed deer with chronic wasting disease, United States*

mpi	96GG genotype							96GS/103NT genotypes					
	No. deer	Tonsil	RAMALT	Urine	Saliva	Feces		No. deer	Tonsil	RAMALT	Urine	Saliva	Feces
6	6	1/6	0/6	ND	0/6	1/5		6	0/6	0/6	ND	0/6	1/2
9	6	3/6	2/6	0/1	2/6	1/4		6	0/6	1/6	0/3	0/6	0/3
12	6	3/6	3/6	0/3	2/6	2/5		6	0/6	1/6	0/6	0/6	0/5
15	6	4/6	4/6	0/1	1/6	4/6		6	1/6	0/6	0/4	0/6	0/4
18	6	6/6	5/6	1/2	1/6	4/5		6	1/6	1/6	0/3	0/6	0/5
21	6	6/6	6/6	1/1	2/5	5/5		6	3/6	1/6	0/5	1/6	0/6
24	5	5/5	5/5	3/4	2/4	4/5		6	5/5	4/6	0/5	1/6	1/4
27	3	3/3	3/3	1/1	1/3	2/3		6	6/6	5/6	1/5	1/6	1/4
29	2	2/2	2/2	1/1	2/2	1/1		6	6/6	6/6	0/5	3/6	1/4
32	1	1/1	1/1	0/0	1/1	1/1		3	3/3	3/3	0/2	0/3	0/2
35	1	1/1	1/1	1/1	1/1	1/1		3	3/3	3/3	0/2	0/3	1/2
39	1	1/1	1/1	1/1	0/1	1/1		3	3/3	3/3	0/3	0/3	2/3
42	0	0	0	0	0	0		2	2/2	2/2	0/2	0/2	1/2
45	0	0	0	0	0	0		2	2/2	2/2	0/2	1/2	1/2
48	0	0	0	0	0	0		2	2/2	2/2	0/2	1/2	1/2
66	0	0	0	0	0	0		1	1/1	1/1	0/1	0/1	0/1

*Values are number of positive samples over total number of samples collected at each timepoint. Tissues and secreta from deer with wild-type genotype 96GG or alternative genotypes 96GS or 103NT of the prion protein gene were compared. mpi, months postinoculation; ND, not done; RAMALT, recto-anal mucosa-associated lymphoid tissue.

### Sample Preparation and CWD Status Analysis

We subjected fecal samples to IOB capture before 4 rounds of sPMCA. We used RT-QuIC as a readout for the sPMCA product; that procedure is hereafter designated as IPQ. In brief, we prepared fecal samples as 10% wt/vol homogenates in 1× phosphate-buffered saline (PBS; 20 mmol/L NaPO_4_, 150 mmol/L NaCl; Sigma-Aldrich, https://www.sigmaaldrich.com). We diluted the homogenates 1:10 to a final volume of 1 mL (100 μL sample:900 μL 1× PBS) and added 2 μL IOB suspension (Bangs Laboratories, Inc., https://www.bangslabs.com) directly to each sample. We incubated the tubes containing the feces/bead mixture by using end-over-end rotation for 30 minutes at room temperature. We transferred each tube to a magnetic separator for 5 minutes, removed the supernatants, and resuspended the demagnetized beads in 10 μL 1× PBS. We added resuspended beads to 90 μL of Tg(CerPrP-E226)5037^+/−^ normal brain homogenate (*37*) and performed 4 rounds of sPMCA. After each round, we froze the sPMCA sample at –20°C until analysis. We prepared 1:100 dilutions of sPMCA samples from rounds 2–4 in 0.1% sodium dodecyl sulfate (SDS; Sigma-Aldrich) and assayed them in quadruplicate (2 μL/well) by using RT-QuIC in >2 plates/sample to achieve a minimum of 8 replicates/sample. We used this method to monitor sequential amplification in each round. We only analyzed round 4 products to determine statistical significance for amyloid seeding activity.

We processed individual saliva and urine samples by using IOB capture and subsequent RT-QuIC, hereafter abbreviated as IQ (*23*). In brief, we added 2 μL IOB suspension to 1 mL of saliva diluted 1:20 in 1× PBS (50 μL saliva:950 μL PBS) or to 1 mL undiluted urine. We placed samples on an end-over-end rotator for 30 minutes at room temperature, transferred the tubes to a magnetic separator for 5 minutes, removed supernatants, and resuspended the demagnetized beads in 10 μL of 0.1% SDS. We added 2 μL of each bead/sample suspension into Greiner Bio-One black optical-bottom microtiter plate wells (VWR, https://www.vwr.com) containing 96 μL RT-QuIC master mix. We assayed a total of 8 replicates/sample in >2 microtiter plates. 

### sPMCA

We performed sPMCA as previously described (*13*). In brief, we combined 90 μL of 10% (wt/vol) Tg(CerPrP-E226)5037^+/−^ normal brain homogenate in 1× PBS containing 1% Triton X-100 (*37*) with 10 μL of each IOB-captured sample in 0.2 mL PCR tubes (ThermoFisher Scientific, https://www.thermofisher.com) containing 2.38 mm and 1.59 mm polytetrafluoroethylene beads (McMaster-Carr, https://www.mcmaster.com). We exposed round 1 sPMCA samples to 30 second pulse sonication followed by 29.5 minutes of rest (1 cycle) for 72 hours (144 cycles total). For rounds 2–4, we added 30 μL of sPMCA product from the previous round to 60 μL of 10% normal brain homogenate and exposed those samples to 24 hours (48 cycles) of the same pulse sonication conditions used for round 1.

### RT-QuIC

We produced, purified, and refolded truncated Syrian hamster recombinant prion protein (rPrP; codons 90–231) as previously described with minor modifications (*15*,*38*,*39*). In brief, we expressed rPrP in *Escherichia coli* BL21-Star cells, harvested inclusion bodies, and solubilized the protein before binding to Ni-agarose resin (GE Healthcare, https://www.gehealthcare.com). We refolded, eluted, and dialyzed the rPRP before aliquoting and storing at 4°C until use.

For RT-QuIC reactions, we loaded each well of a 96-well plate with 96 µL substrate master mix (0.10 mg/mL rPrP, 10 μmol/L thioflavin T [Sigma], 320 mmol/L NaCl [Sigma], 1 mmol/L EDTA [Sigma], and 1× PBS). We diluted each sample in 0.1% SDS and then added 2 μL of the sample to each well. We performed RT-QuIC reactions in a FLUOstar Omega microplate reader (BMG Labtech, https://www.bmglabtech.com) programmed to alternate between 1 minute shaking (double-orbital program at 700 rpm) and 1 minute rest cycles. We measured thioflavin T fluorescence every 15 minutes at 450-nm excitation and 480-nm emission wavelengths and used a fluorescence gain of 1,700. We conducted RT-QuIC experiments at 42°C for 62.5 hours for all IQ studies and 36 hours for all IPQ studies. We displayed RT-QuIC data as 1/lag phase; we defined lag phase as the time (hours) when each replicate fluorescence reached 5 SD above the average baseline fluorescence. We considered samples to be positive if total reaction rate–positive replicates were significantly different (p<0.05) compared with total reaction rate replicates from negative controls by using Mann-Whitney U tests.

### Immunohistochemistry

We confirmed CWD status by using immunohistochemical detection of PrP^CWD^ deposition in tonsil and recto-anal mucosa-associated lymphoid tissue biopsy samples as previously described (*40*). In brief, we treated rehydrated 5 μm tissue sections with 88% formic acid, then citrate buffer for heat-induced epitope retrieval, and blocked with 3% hydrogen peroxide in methanol followed by TNB buffer (0.5% blocking powder; Perkin Elmer, https://www.perkinelmer.com). We incubated slides overnight with monoclonal antibody BAR-224 (1 mg/mL; Cayman Chemical, https://www.caymanchem.com) diluted 1:750 in TNB buffer, followed by Dako Envision+ System horseradish peroxidase–labeled secondary antibody (Agilent, https://www.agilent.com) and 3-amino-9-ethylcarbazole substrate (Abcam, https://www.abcam.com) for visualization. We tested negative control tissues simultaneously in each experiment.

## Results

We report temporal shedding profiles in secreta collected from WTD exposed orally to CWD prion concentrations that might more closely resemble those experienced in nature (*10*). We correlated the shedding profiles with *PRNP* polymorphisms known to modulate CWD infection and disease progression (*34*,*41*).

### Prion Extraction Approach for Feces, Saliva, and Urine

**Figure 1:**
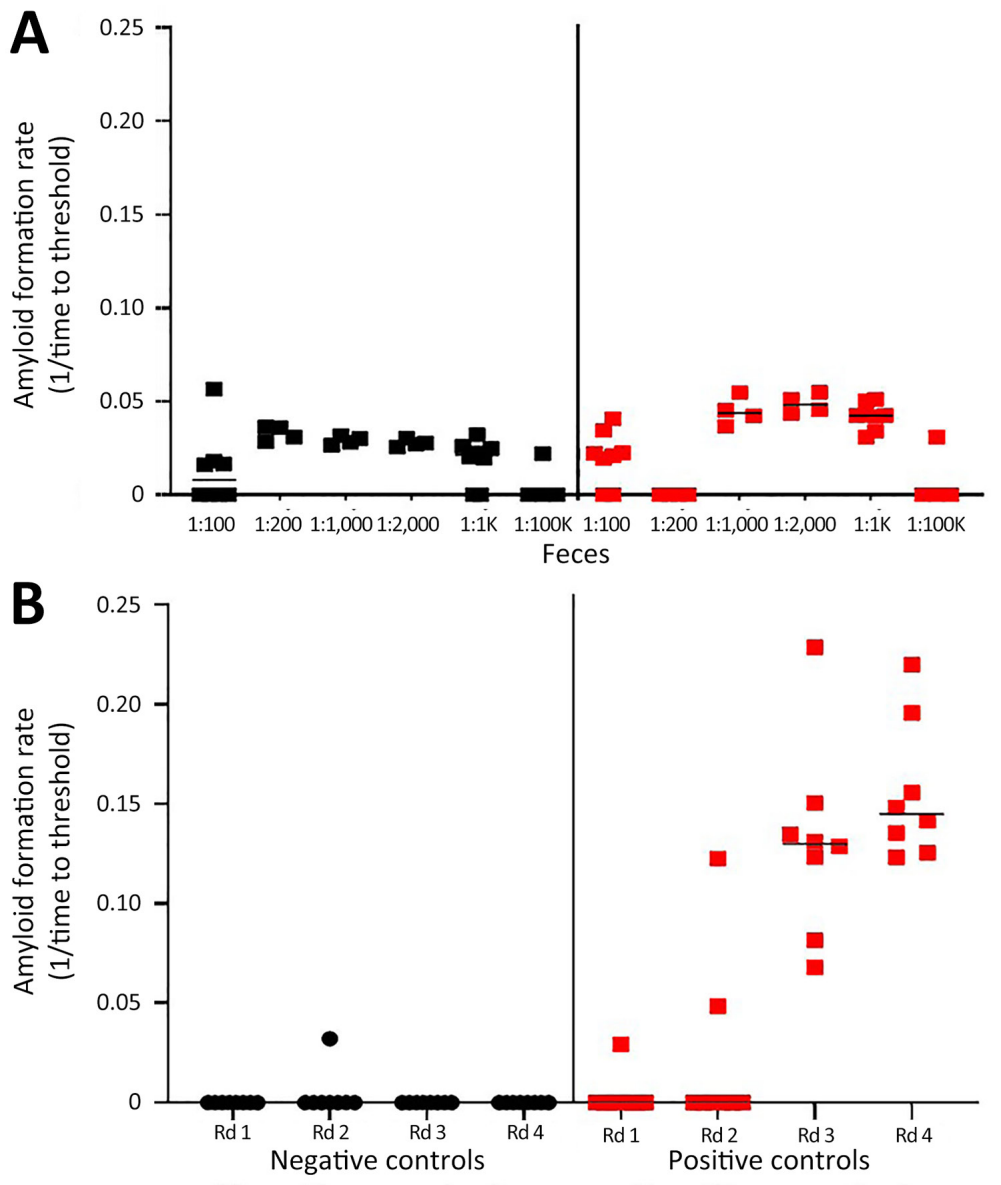
Amyloid formation rates in fecal samples from white-tailed deer with chronic wasting disease in longitudinal study of temporal characterization of prion shedding in secreta, United States. A) Amyloid formation rates measured by using iron oxide bead capture and subsequent real-time quaking-induced conversion. Rates were measured for serial dilutions of fecal samples. B) Amyloid formation rates measured by using iron oxide bead capture, 4 rounds of serial protein misfolding amplification, and subsequent real-time quaking-induced conversion. Rates were measured for 1:100 dilutions of fecal samples from each round of amplification. Black indicates prion-negative feces; red indicates prion-positive feces. Horizontal lines in each grouping indicate median values. Rd, round.

Amplification assays continue to be modified to overcome assay inhibitors or spurious constituents in secreta (*18*,*19*,*21*,*22*). We found that enriching for prions in fecal samples by incorporating IOB capture or phosphotungstic acid precipitation alone often resulted in high nonspecific seeding background in known CWD-negative controls (Figure 1, panel A). To eliminate false-positive backgrounds, fecal samples underwent dilution and IOB capture followed by 4 rounds of sPMCA. The sPMCA products were read out by using RT-QuIC rather than Western blot analysis, thus building upon previous in vitro amplification assay modifications (*13*,*24*). The combination of sPMCA and RT-QuIC readout eliminated false positives in fecal samples and led to enhanced prion seeding activity detection in feces, indicated by higher reaction rates (Figure 1, panel B).

**Figure 2:**
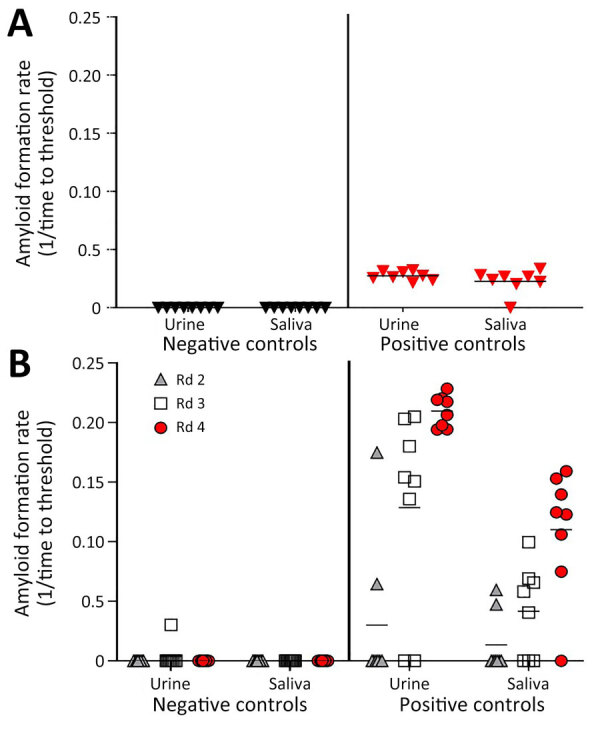
Amyloid formation rates in urine and saliva samples from white-tailed deer with chronic wasting disease in longitudinal study of temporal characterization of prion shedding in secreta, United States. A) Amyloid formation rates measured by using iron oxide bead capture and subsequent real-time quaking-induced conversion. Black indicates prion-negative samples; red indicates prion-positive samples. B) Amyloid formation rates measured by using iron oxide bead capture, 4 rounds of serial protein misfolding amplification, and subsequent real-time quaking-induced conversion in the same samples as those in panel A. Rates were measured for samples after amplification rounds 2–4. Horizontal lines in each grouping indicate median values. Rd, round.

We assayed prion seeding activity in urine and saliva by using IQ and detected positive seeding activity in infected deer compared with negative controls (Figure 2, panel A). Because enhanced detection sensitivity was observed in feces by incorporating IPQ, we used IPQ to measure seeding activity in urine and saliva samples. IQ-positive urine and saliva samples were strongly amplified by using IPQ (Figure 2, panel B). In a separate subset of urine and saliva longitudinal samples, IPQ identified only 1 additional positive saliva sample; the remaining sample results were concordant with IQ results. IPQ did not appreciably increase sensitivity and sPMCA adds 7 days to the analysis protocol. Therefore, we completed analysis of all urine and saliva samples by using IQ.

### Temporal Detection of CWD Shedding in Feces

**Figure 3:**
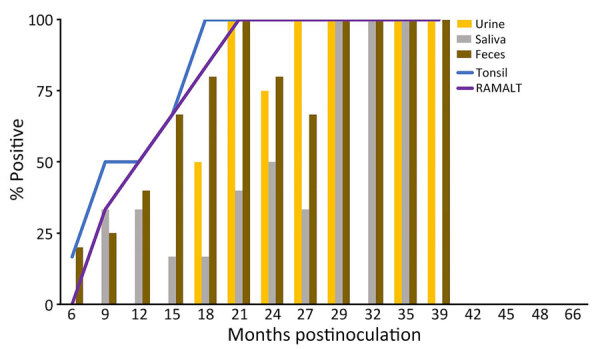
Percentage of prion-positive samples from white-tailed deer with chronic wasting disease that had the prion protein genotype 96GG in study of prion shedding in secreta, United States. Tissue samples and secreta were collected from deer after exposure to low oral doses of chronic wasting disease–positive brain tissue or saliva. RAMALT, recto-anal mucosa-associated lymphoid tissue.

**Figure 4:**
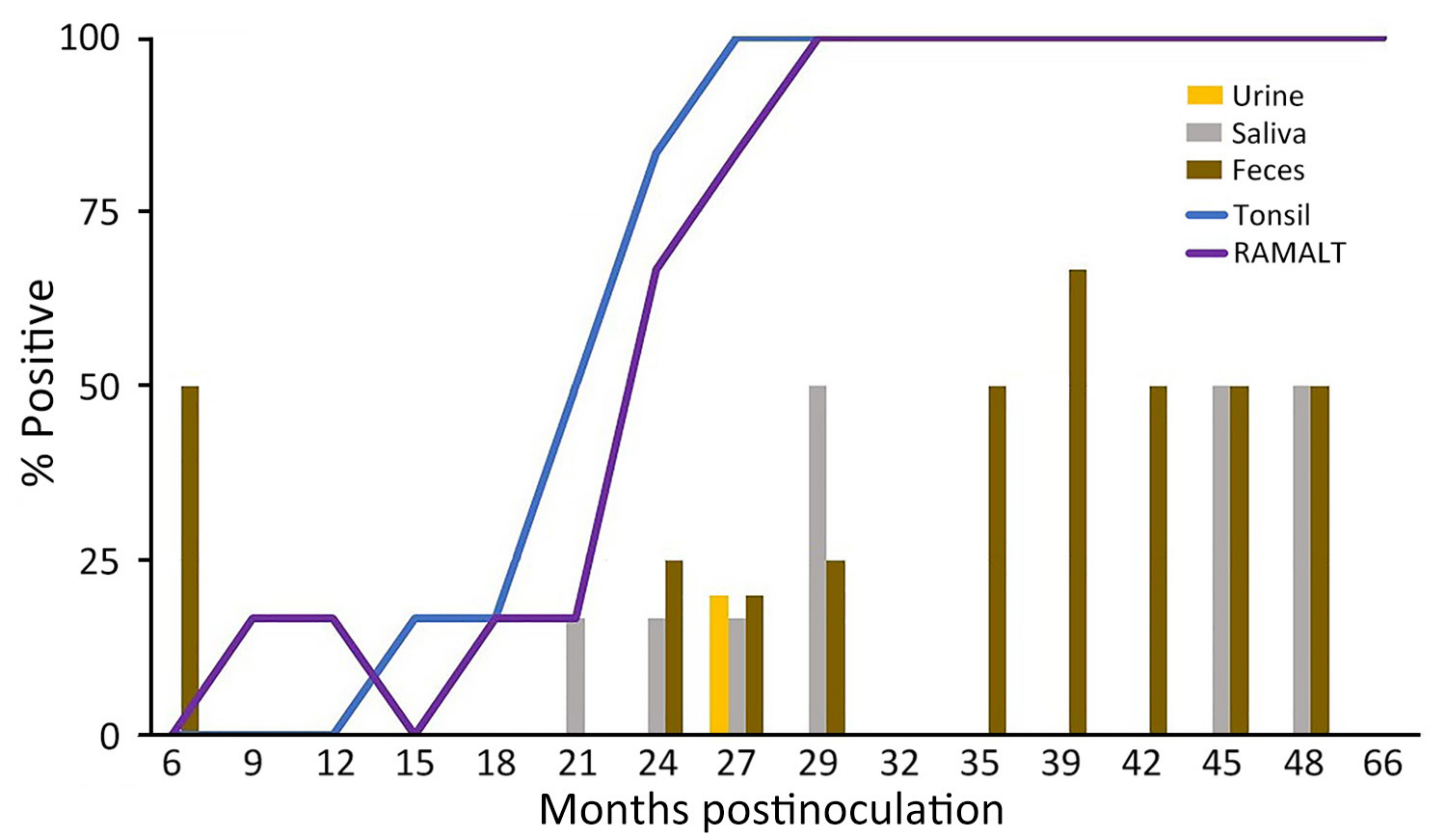
Percentage of prion-positive samples from white-tailed deer with chronic wasting disease that had prion protein genotypes 96GS or 103NT in study of prion shedding in secreta, United States. Tissue samples and secreta were collected from deer after exposure to low oral doses of chronic wasting disease–positive brain tissue or saliva. RAMALT, recto-anal mucosa-associated lymphoid tissue.

We observed CWD prion shedding in feces collected from all 6 deer expressing the 96GG genotype. The earliest detection was at 6 mpi, coinciding with the first positive tonsil biopsy (Table 1; Figure 3). Detectable shedding occurred in all deer within 6 months of the initial RT-QuIC–positive tonsil biopsy (Figure 3). Seeding activity was consistently detected (>4 consecutive positive timepoints) in 4 (66%) of 6 deer and infrequently detected (<3 consecutive positive timepoints) in 2 (33%) of 6 deer. For deer with alternate polymorphisms (96GS or 103NT), >1 positive fecal result was recorded in 4 (66%) of 6 deer during 9–15 months after the first RT-QuIC–positive tonsil biopsy (Table 1; Figure 4). In 1 (17%) of 6 deer, seeding activity was detected in 3 consecutive fecal samples.

Overall, prion shedding was detected in 27 (64%) of 42 fecal samples from the 96GG cohort (Table 2) and 10 (19%) of 52 fecal samples collected from the 96GS/103NT cohort. All 21 fecal samples from sham-inoculated control deer remained negative. Thus, CWD shedding in feces was more frequently detected by IPQ in deer expressing the 96GG genotype than in those expressing alternate polymorphisms (96GS or 103NT).

**Table 2:** Total prion seeding activity detected in each sample type in longitudinal study of temporal characterization of prion shedding in secreta of white-tailed deer with chronic wasting disease, United States*

Sample	96GG genotype	96GS/103NT genotype	Negative controls
Urine	9/16 (56)	1/50 (2)	0/28 (0)
Saliva	15/47 (32)	8/70 (11)	0/28 (0)
Feces	27/42 (64)	10/52 (19)	0/21 (0)

*Values are number of positive samples over total number of samples (%). Secreta from deer with wild-type genotype 96GG or alternative genotypes 96GS or 103NT of the prion protein gene were compared.

### Temporal Detection of CWD Shedding in Urine

CWD prion shedding was observed in urine samples collected from 1 (50%) of 2 deer expressing the 96GG genotype at 18 mpi, 9 months after the first RT-QuIC–positive tonsil biopsy (Table 1; Figure 3). Shedding persisted in 3 (50%) of 6 deer from 18 months until deer were euthanized; 2 (33%) of 6 were positive only at the terminal sample collection. One (17%) of 6 deer had CWD prion–negative urine throughout disease course. Among deer in the 96GS/103NT cohort, CWD prion seeding activity was detected in urine from only 1 (17%) of 6 deer at 27 mpi, coinciding with the first RT-QuIC–positive tonsil biopsy (Table 1; Figure 4).

In total, CWD prion shedding was detected in 9 (56%) of 16 urine samples collected from deer in the 96GG cohort and in 1 (2%) of 50 urine samples collected from deer in the 96GS/103NT cohort (Table 2). All 28 urine samples collected from sham-inoculated control deer remained negative. We show that CWD-infected deer expressing the 96GG genotype shed prions in their urine during later disease stages and did so more frequently and consistently than deer expressing alternate polymorphisms.

### Temporal Detection of CWD Shedding in Saliva

CWD prion shedding occurred in saliva as early as 9 mpi in 2 (33%) of 6 deer expressing the 96GG genotype, coinciding with the first RT-QuIC–positive tonsil biopsy (Table 1; Figure 3). Intermittent shedding occurred throughout the disease course in 5 (83%) of 6 deer during 0–6 months after the first positive tonsil biopsy. In the 96GS/103NT cohort, prion shedding was detected in 3 (50%) of 6 deer starting at 21 mpi, ranging from 3 to 9 months after the first positive tonsil biopsy. Prion detection was infrequent in saliva throughout the disease course (Table 1; Figure 4).

Seeding activity was detected in 15 (32%) of 47 saliva samples collected from deer expressing the 96GG genotype and 8 (11%) of 70 saliva samples collected from deer expressing the 96GS or 103NT genotypes (Table 2). No seeding activity was detected in any of the 28 saliva samples from sham-inoculated control deer. Prion shedding in saliva occurred more frequently than in urine throughout the disease course.

### CWD Initiation and Progression According to WTD *PRNP* Polymorphism

*PRNP* codon 96 polymorphisms are known to affect relative CWD susceptibility and the rate of disease progression in WTD (*10*,*29*,*30*,*42*–*44*). After low-dose oral CWD prion exposure, deer expressing the 96GG genotype had RT-QuIC–positive lymphoid biopsies at 6 (1/6; 17%) to 18 (6/6; 100%) mpi (Table 1; Figure 3). In contrast, deer expressing 96GS or 103NT polymorphisms had delayed CWD-positive status by an additional 9 months; the first RT-QuIC–positive tonsil biopsies occurred at 15–27 mpi (Table 1; Figure 4). Differences in clinical disease kinetics were also observed between the 2 cohorts. All deer with the 96GG genotype were euthanized by 39 mpi because of progressive clinical CWD, whereas 3 (50%) of 6 deer with 96GS or 103NT genotypes remained asymptomatic when euthanized at 48 (n = 2; 96GS) and 66 (n = 1; 103NT) mpi (Table 1). Those results support both slower disease kinetics and reduced prion shedding for WTD expressing the *PRNP* gene encoding 96GS or 103NT polymorphisms.

## Discussion

CWD continues to affect cervid populations in North America, Asia, and Europe (*1*–*4*). The extended asymptomatic phase of disease, during which the infectious CWD prion is shed, appears to be central to environmental contamination and efficient transmission. Yet, little is known about the temporal profiles of CWD shedding in secreta or the role *PRNP* might play in prion shedding. We found consistent prion shedding profiles throughout the disease course (39 mpi) in WTD that had a wild-type *PRNP* genotype at codon 96 (96GG). CWD shedding in secreta was less frequent during the disease course (66 mpi) in deer with alternate polymorphisms (96GS or 103NT), factors that delay CWD infection and progression. Ongoing efforts to establish breeding programs for farmed WTD that have alternate *PRNP* polymorphisms have been initiated (*45*). Our findings suggest that deer expressing alternative *PRNP* polymorphisms might live longer and, although they shed fewer prions throughout CWD course, might over their extended lifespan increase CWD prions in the environment.

We pursued options to improve prion seeding activity detection in fecal samples by combining IOB capture with sPMCA and RT-QuIC readout (IPQ). High-speed centrifugation, sodium phosphotungstic acid, or IOB have previously been used in combination with either PMCA or RT-QuIC to detect prions in feces of CWD-infected animals (*14*,*17*–*23*). However, those using PMCA analyzed products by Western blots rather than RT-QuIC. IPQ eliminated spurious nonspecific amplification that has been noted with fecal samples (*17*,*20*,*22*), providing a much clearer longitudinal CWD fecal shedding profile. We found that the incorporation of IPQ to enhance prion detection sensitivity in urine and saliva samples was not advantageous over the use of IQ.

Feces have been shown to contain low yet sufficient CWD infectivity to initiate infection (*9*,*46*). We observed higher prion concentrations in fecal samples collected from deer expressing the wild-type 96GG genotype than deer expressing alternate polymorphisms. Fecal prions were detected in wild-type deer shortly after biopsy positivity and were consistent throughout disease course, whereas shedding was delayed and inconsistent in deer expressing the alternate polymorphisms. We observed higher prion concentrations in feces than in saliva or urine collected from the 2 deer cohorts, possibly suggesting the prion load in feces is higher than previously recognized (*18*,*21*) or IPQ removes inhibitors that permit enhanced detection in feces not attained in urine or saliva samples Further assay development will be needed to address this question and to establish prion titers across all 3 secreta types.

Bioassay studies in WTD and cervid PrP–expressing transgenic mice have revealed low levels of prion infectivity in urine and saliva collected at various stages of CWD (*8*–*10*,*47*). In vitro detection has been challenging, presumably because of low concentrations or inhibitors in those bodily secretions (*11*–*13*,*15*,*16*,*23*). By combining IOB capture and RT-QuIC amplification, we found consistent CWD prion shedding in urine and saliva collected from WTD expressing wildtype *PRNP* compared with those expressing alternate *PRNP* polymorphisms. Our findings support an earlier study that showed CWD shedding in urine occurred less frequently in deer with more CWD-resistant genotypes (*18*).

Overall, our results support the tenet that prion shedding in secreta occurs more frequently and consistently in WTD expressing *PRNP* genotype 96GG and less frequently in deer with *PRNP* 96GS or 103NT polymorphisms. We also found more consistent CWD prion shedding in saliva than urine for both cohorts, suggesting that saliva might be a plausible vector for efficient disease transmission. This finding reinforces previous studies reporting that saliva is more infectious than urine or feces after experimental CWD prion inoculation (*8*,*10*,*47*,*48*). It would be advantageous to determine which secreta is responsible for the efficient CWD transmission dynamics. However, many variables exist that might influence infection, such as infection route, genotype, environmental/soil factors, CWD prion strain, and dose, and it might be misleading to suggest one secretum contributes more to CWD spread than another.

In conclusion, our findings indicate the role prion shedding might have on CWD transmission, particularly given the presumed low prion content in secreta. We conducted our study with an overarching eye on the potential advances, limitations, and opportunities that remain to fully exploit ultrasensitive prion seeding assays used to examine amyloid development in complex milieus and matrices. Our findings demonstrate improved amplification methods can be used to identify early antemortem CWD prion shedding in cervids that might aid in disease surveillance. 

## References

[1] Sohn HJ, Kim JH, Choi KS, Nah JJ, Joo YS, Jean YH, et al. A case of chronic wasting disease in an elk imported to Korea from Canada. J Vet Med Sci. 2002;64:855–8. 10.1292/jvms.64.855 12399615

[2] Benestad SL, Mitchell G, Simmons M, Ytrehus B, Vikøren T. First case of chronic wasting disease in Europe in a Norwegian free-ranging reindeer. Vet Res. 2016;47:88. 10.1186/s13567-016-0375-4 27641251 PMC5024462

[3] Pirisinu L, Tran L, Chiappini B, Vanni I, Di Bari MA, Vaccari G, et al. Novel type of chronic wasting disease detected in moose (*Alces alces*), Norway. Emerg Infect Dis. 2018;24:2210–8. 10.3201/eid2412.180702 30457526 PMC6256397

[4] US Geological Survey, National Wildlife Health Center. Expanding distribution of chronic wasting disease. 2022 [cited 2024 Jun 1]. https://www.usgs.gov/centers/nwhc/science/expanding-distribution-chronic-wasting-disease

[5] Miller MW, Williams ES. Prion disease: horizontal prion transmission in mule deer. Nature. 2003;425:35–6. 10.1038/425035a 12955129

[6] Miller MW, Williams ES, Hobbs NT, Wolfe LL. Environmental sources of prion transmission in mule deer. Emerg Infect Dis. 2004;10:1003–6. 10.3201/eid1006.040010 15207049 PMC3323154

[7] Mathiason CK, Hays SA, Powers J, Hayes-Klug J, Langenberg J, Dahmes SJ, et al. Infectious prions in pre-clinical deer and transmission of chronic wasting disease solely by environmental exposure. PLoS One. 2009;4:e5916. 10.1371/journal.pone.0005916 19529769 PMC2691594

[8] Mathiason CK, Powers JG, Dahmes SJ, Osborn DA, Miller KV, Warren RJ, et al. Infectious prions in the saliva and blood of deer with chronic wasting disease. Science. 2006;314:133–6. 10.1126/science.1132661 17023660

[9] Haley NJ, Mathiason CK, Zabel MD, Telling GC, Hoover EA. Detection of sub-clinical CWD infection in conventional test-negative deer long after oral exposure to urine and feces from CWD+ deer. PLoS One. 2009;4:e7990. 10.1371/journal.pone.0007990 19956732 PMC2776529

[10] Denkers ND, Hoover CE, Davenport KA, Henderson DM, McNulty EE, Nalls AV, et al. Very low oral exposure to prions of brain or saliva origin can transmit chronic wasting disease. PLoS One. 2020;15:e0237410. 10.1371/journal.pone.0237410 32817706 PMC7446902

[11] John TR, Schätzl HM, Gilch S. Early detection of chronic wasting disease prions in urine of pre-symptomatic deer by real-time quaking-induced conversion assay. Prion. 2013;7:253–8. 10.4161/pri.24430 23764839 PMC3783112

[12] Rubenstein R, Chang B, Gray P, Piltch M, Bulgin MS, Sorensen-Melson S, et al. Prion disease detection, PMCA kinetics, and IgG in urine from sheep naturally/experimentally infected with scrapie and deer with preclinical/clinical chronic wasting disease. J Virol. 2011;85:9031–8. 10.1128/JVI.05111-11 21715495 PMC3165845

[13] Davenport KA, Hoover CE, Denkers ND, Mathiason CK, Hoover EA. Modified protein misfolding cyclic amplification overcomes real-time quaking-induced conversion assay inhibitors in deer saliva to detect chronic wasting disease prions. J Clin Microbiol. 2018;56:e00947–18. 10.1128/JCM.00947-18 29950332 PMC6113454

[14] Pulford B, Spraker TR, Wyckoff AC, Meyerett C, Bender H, Ferguson A, et al. Detection of PrP^CWD^ in feces from naturally exposed Rocky Mountain elk (*Cervus elaphus nelsoni*) using protein misfolding cyclic amplification. J Wildl Dis. 2012;48:425–34. 10.7589/0090-3558-48.2.425 22493117

[15] Henderson DM, Manca M, Haley NJ, Denkers ND, Nalls AV, Mathiason CK, et al. Rapid antemortem detection of CWD prions in deer saliva. PLoS One. 2013;8:e74377. 10.1371/journal.pone.0074377 24040235 PMC3770611

[16] Henderson DM, Denkers ND, Hoover CE, Garbino N, Mathiason CK, Hoover EA. Longitudinal detection of prion shedding in saliva and urine by chronic wasting disease–infected deer by real-time quaking-induced conversion. J Virol. 2015;89:9338–47. 10.1128/JVI.01118-15 26136567 PMC4542351

[17] Henderson DM, Tennant JM, Haley NJ, Denkers ND, Mathiason CK, Hoover EA. Detection of chronic wasting disease prion seeding activity in deer and elk feces by real-time quaking-induced conversion. J Gen Virol. 2017;98:1953–62. 10.1099/jgv.0.000844 28703697 PMC5817259

[18] Plummer IH, Wright SD, Johnson CJ, Pedersen JA, Samuel MD. Temporal patterns of chronic wasting disease prion excretion in three cervid species. J Gen Virol. 2017;98:1932–42. 10.1099/jgv.0.000845 28708047

[19] Cheng YC, Hannaoui S, John TR, Dudas S, Czub S, Gilch S. Real-time quaking-induced conversion assay for detection of CWD prions in fecal material. J Vis Exp. 2017;127:56373. 28994814 10.3791/56373 PMC5752360

[20] Cheng YC, Hannaoui S, John TR, Dudas S, Czub S, Gilch S. Early and non-invasive detection of chronic wasting disease prions in elk feces by real-time quaking induced conversion. PLoS One. 2016;11:e0166187. 10.1371/journal.pone.0166187 27829062 PMC5102397

[21] Tennant JM, Li M, Henderson DM, Tyer ML, Denkers ND, Haley NJ, et al. Shedding and stability of CWD prion seeding activity in cervid feces. PLoS One. 2020;15:e0227094. 10.1371/journal.pone.0227094 32126066 PMC7053746

[22] Hwang S, Greenlee JJ, Nicholson EM. Real-time quaking-induced conversion detection of PrP^Sc^ in fecal samples from chronic wasting disease infected white-tailed deer using bank vole substrate. Front Vet Sci. 2021;8:643754. 10.3389/fvets.2021.643754 33748218 PMC7969510

[23] Denkers ND, Henderson DM, Mathiason CK, Hoover EA. Enhanced prion detection in biological samples by magnetic particle extraction and real-time quaking-induced conversion. J Gen Virol. 2016;97:2023–9. 10.1099/jgv.0.000515 27233771 PMC5903251

[24] McNulty EE, Nalls AV, Xun R, Denkers ND, Hoover EA, Mathiason CK. *In vitro* detection of haematogenous prions in white-tailed deer orally dosed with low concentrations of chronic wasting disease. J Gen Virol. 2020;101:347–61. 10.1099/jgv.0.001367 31846418 PMC7416609

[25] Henderson DM, Davenport KA, Haley NJ, Denkers ND, Mathiason CK, Hoover EA. Quantitative assessment of prion infectivity in tissues and body fluids by real-time quaking-induced conversion. J Gen Virol. 2015;96:210–9. 10.1099/vir.0.069906-0 25304654 PMC4268821

[26] Ferreira NC, Charco JM, Plagenz J, Orru CD, Denkers ND, Metrick MA II, et al. Detection of chronic wasting disease in mule and white-tailed deer by RT-QuIC analysis of outer ear. Sci Rep. 2021;11:7702. 10.1038/s41598-021-87295-8 33833330 PMC8032746

[27] Arifin MI, Hannaoui S, Chang SC, Thapa S, Schatzl HM, Gilch S. Cervid prion protein polymorphisms: role in chronic wasting disease pathogenesis. Int J Mol Sci. 2021;22:2271. 10.3390/ijms22052271 33668798 PMC7956812

[28] O’Rourke KI, Besser TE, Miller MW, Cline TF, Spraker TR, Jenny AL, et al. PrP genotypes of captive and free-ranging Rocky Mountain elk (*Cervus elaphus nelsoni*) with chronic wasting disease. J Gen Virol. 1999;80:2765–2679. 10.1099/0022-1317-80-10-2765 10573173

[29] Johnson C, Johnson J, Vanderloo JP, Keane D, Aiken JM, McKenzie D. Prion protein polymorphisms in white-tailed deer influence susceptibility to chronic wasting disease. J Gen Virol. 2006;87:2109–14. 10.1099/vir.0.81615-0 16760415

[30] Johnson CJ, Herbst A, Duque-Velasquez C, Vanderloo JP, Bochsler P, Chappell R, et al. Prion protein polymorphisms affect chronic wasting disease progression. PLoS One. 2011;6:e17450. 10.1371/journal.pone.0017450 21445256 PMC3060816

[31] Jewell JE, Conner MM, Wolfe LL, Miller MW, Williams ES. Low frequency of PrP genotype 225SF among free-ranging mule deer (*Odocoileus hemionus*) with chronic wasting disease. J Gen Virol. 2005;86:2127–34. 10.1099/vir.0.81077-0 16033959

[32] Hamir AN, Gidlewski T, Spraker TR, Miller JM, Creekmore L, Crocheck M, et al. Preliminary observations of genetic susceptibility of elk (*Cervus elaphus nelsoni*) to chronic wasting disease by experimental oral inoculation. J Vet Diagn Invest. 2006;18:110–4. 10.1177/104063870601800118 16566268

[33] Kraft CN, Denkers ND, Mathiason CK, Hoover EA. Longitudinal detection of prion shedding in nasal secretions of CWD-infected white-tailed deer. J Gen Virol. 2023;104:001825. 10.1099/jgv.0.001825 36748533 PMC10233467

[34] Johnson C, Johnson J, Clayton M, McKenzie D, Aiken J. Prion protein gene heterogeneity in free-ranging white-tailed deer within the chronic wasting disease affected region of Wisconsin. J Wildl Dis. 2003;39:576–81. 10.7589/0090-3558-39.3.576 14567218

[35] Robinson SJ, Samuel MD, O’Rourke KI, Johnson CJ. The role of genetics in chronic wasting disease of North American cervids. Prion. 2012;6:153–62. 10.4161/pri.19640 22460693 PMC7082092

[36] Henderson DM, Denkers ND, Hoover CE, McNulty EE, Cooper SK, Bracchi LA, et al. Progression of chronic wasting disease in white-tailed deer analyzed by serial biopsy RT-QuIC and immunohistochemistry. PLoS One. 2020;15:e0228327. 10.1371/journal.pone.0228327 32059005 PMC7021286

[37] Angers RC, Seward TS, Napier D, Green M, Hoover E, Spraker T, et al. Chronic wasting disease prions in elk antler velvet. Emerg Infect Dis. 2009;15:696–703. 10.3201/eid1505.081458 19402954 PMC2687044

[38] Wilham JM, Orrú CD, Bessen RA, Atarashi R, Sano K, Race B, et al. Rapid end-point quantitation of prion seeding activity with sensitivity comparable to bioassays. PLoS Pathog. 2010;6:e1001217. 10.1371/journal.ppat.1001217 21152012 PMC2996325

[39] Atarashi R, Sano K, Satoh K, Nishida N. Real-time quaking-induced conversion: a highly sensitive assay for prion detection. Prion. 2011;5:150–3. 10.4161/pri.5.3.16893 21778820 PMC3226039

[40] Denkers ND, Hayes-Klug J, Anderson KR, Seelig DM, Haley NJ, Dahmes SJ, et al. Aerosol transmission of chronic wasting disease in white-tailed deer. J Virol. 2013;87:1890–2. 10.1128/JVI.02852-12 23175370 PMC3554158

[41] O’Rourke KI, Spraker TR, Hamburg LK, Besser TE, Brayton KA, Knowles DP. Polymorphisms in the prion precursor functional gene but not the pseudogene are associated with susceptibility to chronic wasting disease in white-tailed deer. J Gen Virol. 2004;85:1339–46. 10.1099/vir.0.79785-0 15105552

[42] Otero A, Duque Velásquez C, Johnson C, Herbst A, Bolea R, Badiola JJ, et al. Prion protein polymorphisms associated with reduced CWD susceptibility limit peripheral PrP^CWD^ deposition in orally infected white-tailed deer. BMC Vet Res. 2019;15:50. 10.1186/s12917-019-1794-z 30717795 PMC6360794

[43] Race B, Meade-White K, Miller MW, Fox KA, Chesebro B. In vivo comparison of chronic wasting disease infectivity from deer with variation at prion protein residue 96. J Virol. 2011;85:9235–8. 10.1128/JVI.00790-11 21697479 PMC3165848

[44] Hoover CE, Davenport KA, Henderson DM, Denkers ND, Mathiason CK, Soto C, et al. Pathways of prion spread during early chronic wasting disease in deer. J Virol. 2017;91:e00077–17. 10.1128/JVI.00077-17 28250130 PMC5411598

[45] Haley N, Donner R, Merrett K, Miller M, Senior K. Selective breeding for disease-resistant *PRNP* variants to manage chronic wasting disease in farmed whitetail deer. Genes (Basel). 2021;12:1396. 10.3390/genes12091396 34573378 PMC8471411

[46] Tamgüney G, Miller MW, Wolfe LL, Sirochman TM, Glidden DV, Palmer C, et al. Asymptomatic deer excrete infectious prions in faeces. Nature. 2009;461:529–32. 10.1038/nature08289 19741608 PMC3186440

[47] Haley NJ, Seelig DM, Zabel MD, Telling GC, Hoover EA. Detection of CWD prions in urine and saliva of deer by transgenic mouse bioassay. PLoS One. 2009;4:e4848. 10.1371/journal.pone.0004848 19293928 PMC2654070

[48] Tamgüney G, Richt JA, Hamir AN, Greenlee JJ, Miller MW, Wolfe LL, et al. Salivary prions in sheep and deer. Prion. 2012;6:52–61. 10.4161/pri.6.1.16984 22453179 PMC3338966

